# The contribution of childhood trauma to irritability symptoms

**DOI:** 10.1002/jcv2.12260

**Published:** 2024-07-01

**Authors:** Lana Ruvolo Grasser, Ruiyu Yang, Melissa A. Brotman, Jillian Lee Wiggins

**Affiliations:** ^1^ Neuroscience and Novel Therapeutics Unit Emotion and Development Branch National Institute of Mental Health National Institutes of Health Bethesda Maryland USA; ^2^ San Diego State University / University of California San Diego Joint Doctoral Program in Clinical Psychology San Diego California USA; ^3^ Department of Psychology San Diego State University San Diego California USA

**Keywords:** abuse, child and adolescent, deprivation, multiple regression, neglect, threat

## Abstract

**Background:**

Irritability is a transdiagnostic psychiatric phenotype defined as an increased proneness to anger relative to peers. Trauma is defined as actual or threatened death, serious injury, or sexual violence while adversity more broadly describes difficult or challenging situations including abuse, neglect, and household dysfunction. Irritability [or aggression] is symptom of posttraumatic stress disorder (PTSD) and may arise in response to trauma or traumatic events. Responses to negative early life experiences may differ based on the type of exposure, for example, threat (abuse) versus deprivation (neglect), with implications for development of psychopathology. Therefore, the objective of this study was to investigate the relation between exposure to threat and deprivation, and irritability in a predominantly Hispanic/Latin sample. We hypothesized unique effects of threat versus deprivation on irritability.

**Methods:**

We investigated relations between threat and deprivation aspects of childhood trauma (within each dimension) and later irritability in a sample of *n* = 48 (26F) youth ages 9–19 (*M*_*age*_ = 14.89, *SD* = 2.04) recruited based on trauma exposure. Multivariate regression tested the unique effects of threat and deprivation (measurement: Childhood Trauma Questionnaire) on irritability (measurement: Affective Reactivity Index).

**Results:**

Greater threat exposure was associated with more severe self‐reported irritability, *F*(1,46) = 8.64, *B* = 0.40, *R* ^2^ = 0.14, *p* = 0.005. Findings remained significant after controlling for values of excessive influence and the non‐significant effect of gender (*B* = 0.25, *t* = 1.88, *p* = 0.067). When looking at the unique effects of threat adjusted for deprivation, the relation between threat and irritability remained significant, *B* = 0.35, *t* = 2.45, *p* = 0.019. There was no significant association between deprivation and irritability, *F*(1,46) = 3.35, *B* = 0.26, *R* ^2^ = 0.05, *p* = 0.074.

**Conclusions:**

Exposure to threat, but not deprivation, may increase risk for irritability in youth. Early life experiences should be considered in assessment and treatment of youth with clinically impairing irritability. Transdiagnostic treatments targeting irritability should be tested for youth with trauma exposure who do not meet criteria for post‐traumatic stress disorder.

## Graphical abstract

Grasser et al. find unique effects of exposure to threat during childhood, but not deprivation, driving associations between cumulative trauma and irritability in a sample of predominantly Hispanic/Latin youth.

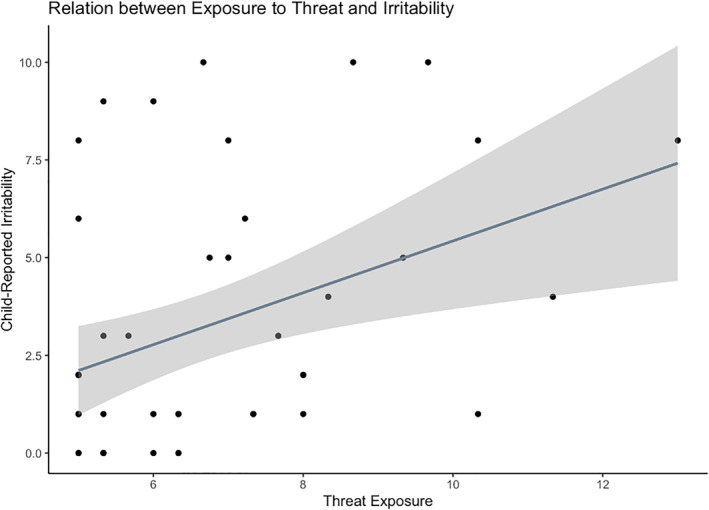

**Key points**

- What's Known
  o Irritability is a common, transdiagnostic phenotype
  o Irritability is a symptom of posttraumatic stress disorder, as part of Cluster E—Arousal and Reactivity
- What's New
  o We provide evidence in youth that exposure to trauma is associated with irritability as a *construct* defined by multiple items, not just a single *symptom* that is characteristic of other constructs, like PTSD
  o Threat, but not deprivation, drove the relation between trauma exposure and irritability in youth
- What's Relevant
  o This work calls for transdiagnostic treatment approaches for trauma‐exposed youth and further exploration into trauma‐informed treatment approaches like exposure‐based cognitive behavioral therapy for trauma‐exposed youth who may not endorse full criteria for PTSD but may endorse other clinically significant symptoms like irritability

## INTRODUCTION

Early life experiences can alter the socioemotional developmental trajectory (Wiggins & Monk, 2013). Unfortunately, traumatic early life experiences—that is, actual or threatened death, serious injury, and/or sexual violence (“Diagnostic and statistical manual of mental disorders: DSM‐5™, 5th ed,” 2013)—and adversity—more broadly including deprivation, neglect, abuse, and household dysfunction (Felitti et al., 1998)—are pervasive; estimates indicate that 20%–60% of youth are exposed before the age of 18 (Breslau et al., 2004). Adverse experiences and traumatic events may contribute to the development, presentation, maintenance, and severity of clinical symptoms, including irritability and aggression (“Diagnostic and statistical manual of mental disorders: DSM‐5™, 5th ed,” 2013). Irritability is defined as an increased proneness to anger relative to peers (Brotman et al., 2017a). Irritability is transdiagnostic, presenting in several psychiatric conditions (including posttraumatic stress, anxiety, and depression) and is one of the most common reasons youth present for clinical care (Brotman et al., 2017a, 2017b; Leibenluft & Stoddard, 2013; Stringaris, 2011; Stringaris et al., 2009). Critically, irritability may arise in response to traumatic events (Henriksen et al., 2021), and elevated irritability is included as a symptom of posttraumatic stress disorder (PTSD) as part of Cluster E, arousal and reactivity, in the DSM‐5 ("Diagnostic and statistical manual of mental disorders: DSM‐5™, 5th ed,” 2013). However, there is a paucity of research evaluating irritability as a clinical consequences of negative early life experiences such as trauma and adversity.

Responses to negative early life experiences may differ based on the number and type(s) of exposure, and resultant symptoms may vary in terms of presentation, intensity, and duration (Hinchey, Grasser, et al., 2023). Two models have furthered understanding of the impact of negative early life experiences on mental and physical health outcomes: (a) the cumulative risk model (Evans et al., 2013), and (b) the dimensional model of childhood adversity (McLaughlin et al., 2014; Sheridan & McLaughlin, 2014). The cumulative risk model describes risk for negative outcomes as increasing with the cumulative number of exposures to any adverse/traumatic event. Indeed, cumulative exposures are broadly associated with increasing risk for and severity of a wide variety of mental and physical health problems (Hughes et al., 2017). By contrast, the dimensional model considers the impact of types of exposures, that is, threat and deprivation, on behavioral and clinical outcomes (McLaughlin et al., 2014; McLaughlin & Sheridan, 2016).

Here, threat is defined as experiences involving harm or threat of harm (e.g., community violence, domestic violence, physical, verbal, and sexual abuse) while deprivation is defined as experiences involving absence of expected inputs from the environment that are necessary for health development (e.g., poverty, neglect, lack of positive caregiver interaction or caregiver separation) (Machlin et al., 2019; McLaughlin & Sheridan, 2016; Miller et al., 2021). Youths may endorse greater severity of one dimension compared to the other, and such varying clinical presentations may represent different etiologies (Wiggins & Monk, 2013), which may in turn inform prognosis and treatment.

In support of this dimensional model, exposure to deprivation is commonly implicated in deficits in executive functioning, cognitive abilities, and verbal skills (Schäfer et al., 2023) while exposure to threat is commonly associated with internalizing and externalizing psychopathology (Busso et al., 2017; Henry et al., 2021; Miller et al., 2018). For example, threats may interfere with goal attainment, eliciting frustration, which can lead to both anger and aggression—all of which are characteristic of irritability (Brotman et al., 2017a). Taken together, while exposure to increasing number of negative early life experiences may be associated with greater severity of psychopathology, the *type* of trauma exposure (e.g., threat vs. deprivation), may be more specifically correlated with the types of deficits experienced afterward, for example, clinically impairing irritability, which spans both internalizing and externalizing psychopathology.

Despite the codification of irritability as a PTSD symptom, there have been few studies specifically evaluating the impact of cumulative trauma and trauma subtypes on irritability as a psychiatric construct. Indeed, of the little extant work on trauma as an etiological contributor to irritability that has been done, most studies have been limited by single‐item assessments of irritability that do not fully capture irritability as a transdiagnostic psychiatry construct (Tseng et al., 2023), but have provided foundational evidence on the positive correlation between trauma exposure and irritability for the proposed hypotheses (Bilgi et al., 2017; Henriksen et al., 2021; McCoy et al., 2014).

Previous work in adults has emphasized the irritability component of the hyperarousal cluster of PTSD and the overlap between irritability, emotion regulation difficulties, and other PTSD symptoms (Villalta et al., 2018; Zhan et al., 2023). Testing the relation between trauma and irritability in youth is important given that many characterization and treatment studies of irritability in youth exclude exposure to trauma, potentially biasing findings if indeed greater severity of irritability as a construct, not just a singular item/criterion, is correlated with higher rates of exposure. One study, for example, suggests that treatment of disruptive behavior disorders in youth with a history of trauma exposure may reduce both irritability and other trauma‐related symptoms (Hwang et al., 2021). Additionally, the study of irritability has been plagued by an overarching problem in the field of psychology, that is the diversity of included participants and the generalizability of the literature (Henrich et al., 2010). Given that prior work has been biased toward white, middle‐class samples, we focused here on a lower socioeconomic status, predominantly Hispanic/Latin sample to balance the overall generalizability of the literature. Therefore, the aim of this study was to investigate the relation between exposure to negative early life experiences and irritability in a sample of adolescents who predominantly identified as Hispanic/Latin. We tested two connected hypotheses: (a) cumulative exposure is associated with irritability, such that youth with greater exposure endorse greater severity of symptoms, and (b) there are unique effects of threat versus deprivation on irritability.

## METHODS

### Participants

We leveraged data from two previously described cohorts of treatment‐seeking youth (Schwartz et al., 2019; Yang et al., 2023) (*N* = 49) between the ages of 9 and 19 (see Table 1 for demographic characteristics). Of note, youth and their families who seek treatment may likely experience elevated psychological symptoms, including elevated irritability, compared to that of the general population (e.g., Amos Nwankwo et al., 2021). Indeed, irritability is one of the most common reasons driving youth and their families to seek psychiatric care (Brotman et al., 2017a, 2017b). This could, in part, stem from cumulative effects of trauma and stress, underscoring the importance of studying this population of youth. The sample and findings described herein should be contextualized with this in mind. Participants in both cohorts were recruited from the San Diego area and represented a predominantly Hispanic/Latin and/or low‐income population. Participants had varying profiles of childhood adversity. One participant was excluded from analyses as they were missing the primary outcome measure, resulting in a final sample of *n* = 48. *N* = 31 were seeking trauma‐focused cognitive behavioral therapy (but had not yet begun); *n* = 17 were seeking anxiety‐ and depression‐focused cognitive behavioral therapy (but were in the control group). All behavioral data for the research described herein was collected prior to therapy. Samples did not differ by cohort, see Supplementary Table 1.

**TABLE 1:** Sociodemographic characteristics of participants and descriptive statistics.

	Boys (*n* = 22)		Girls (*n* = 26)		Full sample (*n* = 48)	
	*n*	%	*n*	%	*n*	%
Cohort						
A	9	40.9	8	30.8	17	35.4
B	13	59.1	18	69.2	31	64.6
Race^a^						
Black	2	11.8	3	13.6	5	12.8
Asian/Pacific Islander	1	5.9	1	4.5	2	5.1
White	7	41.2	8	36.4	15	7
Biracial	1	5.9	2	9.1	3	7.7
Other	6	35.3	8	36.4	14	35.9
Ethnicity						
Hispanic/Latin	13	59.1	19	73.1	32	66.7
Not Hispanic/Latin	9	40.9	7	26.9	16	33.3

	*M*	*SD*	*M*	*SD*	*M*	*SD*
Threat exposure	6.29	1.44	6.95	2.22	6.65	1.91
Deprivation exposure	8.50	2.39	8.68	3.32	8.60	2.90
Cumulative exposure	7.18	1.61	7.64	2.28	7.43	2.00
Irritability, child report	2.14	2.27	4.12	3.59	3.21	3.19
Irritability, parent report	2.72	3.15	4.05	3.85	3.45	3.57

*Note*: Participants were on average 14.89 years old (*SD* = 2.04), and participant age did not differ by gender (*M*_*boys*_ = 14.73, *SD* = 2.38; *M*_*girls*_ = 15.01, *SD* = 1.73; *t*(37.69) = ‐0.46, *p* = 0.647, *d* = −0.15, 95% CIs_std_ = [LLCI: −0.79, ULCI: 0.49]).

^a^ Reflects the number and percentage of participants answering “yes” to this question. Self‐reported race data was missing for *n* = 9. No participants reported being Native American or Alaskan Native.

### Ethical considerations

Procedures were approved by the University of California, San Diego Institutional Review Board and were accepted by joint agreement at San Diego State University. Parents gave written informed consent and permission for their children to participate, and children provided written assent.

### Measures

Irritability over the last 6 months was measured using the Affective Reactivity Index (ARI) (Haller et al., 2020; Stringaris et al., 2012) youth self‐report. The ARI is a seven‐item measure containing six symptom items (regarding mood, anger, and temper outbursts) and one impairment item. Responses range from 0 (‘not true’) to 2 (‘certainly true’) for possible scores ranging from 0 to 12 (Stringaris et al., 2012). Cronbach's alpha for the ARI child report was excellent, *α* = 0.89. Corresponding parent‐report of irritability was obtained (see Supplementary Materials); items are identical for measuring irritability over the last 6 months across parent and child versions. Cronbach's alpha for the ARI parent report was excellent, *α* = 0.91. Consistent with previous publications (Hodgdon et al., 2021), we used youth self‐report as the primary indicator given that adolescents' insights into their own affective state represent a separate construct from parent‐reported irritability (Dougherty et al., 2021). Spanish versions of the questionnaires were provided when the parent/caregiver's primary/preferred language was Spanish.

The Childhood Trauma Questionnaire (CTQ) (Bernstein et al., 1998) was used to assess exposure to trauma by youth self‐report. The Short Form includes 28 items, 3 of which are validity items detecting underreporting of maltreatment. Responses range from 1 (‘never true’) to 5 (‘very often true’) to capture both whether an exposure occurred and the frequency of the exposure (Bernstein et al., 2003). The CTQ contains five subscales capturing experiences of physical, emotion, and sexual abuse as well as physical and emotional neglect; each subscale score can range from 5 to 25 (Bernstein et al., 2003). The threat composite score was defined as the average of scores on the CTQ emotional, physical, and sexual abuse subscales, as previously described (Banihashemi et al., 2021, 2022; Blair et al., 2019). The deprivation composite score was defined as the average of scores on the emotional and physical neglect sections, as previously described (Banihashemi et al., 2021, 2022; Blair et al., 2019). Validation of this structure has previously been reported using confirmatory factor analysis (Greene et al., 2021). Cumulative trauma was defined as the average of scores across all five sections. Further details regarding the CTQ and its psychometric properties can be found in the Supplementary Materials.

### Data screening

Item‐level data were screened for out‐of‐range values, and no such values were identified. Univariate outliers were not eliminated or corrected in order to maximize the sample size given the limited sample, and to accurately represent the full spectrum of symptoms from normative to clinically impairing and exposures from none to severe—represented by ‘outlier’ cases. However, for all regression analyses, values of excessive influence were inspected based on Cook's distance >0.05. Results for all analyses are presented herein for raw data, as well as data corrected for values of excessive influence whereby such values were eliminated. Little's MCAR was used to assess the structure of missing data, and Little's *χ* ^2^ = 25.40, *p* = 0.115 confirmed that data were missing completely at random. The only variable with missingness was the ARI parent report (4.17% missing). Therefore, we chose to impute missing data using eight iterations of multiple imputation. Previous research has identified a moderating role of sex on relations between irritability and internalizing/externalizing symptoms (Humphreys et al., 2019), as well as higher levels of chronic and episodic irritability in girls compared to boys (Leibenluft et al., 2006). Additionally, in some samples, frequency of adverse exposures varies by sex and may be differentially associated with psychopathology by sex (Prachason et al., 2023). Therefore, we assessed sex differences (male vs. female) in study variables and include gender as a covariate in regression models. We did not include age as a covariate given that age and adverse exposures may be difficult to disentangle from one another as individuals have more opportunities to be exposed with more years of life and potentially greater environmental exploration (Grasser, Saad, et al., 2023). In our sample age and exposure were not significantly correlated (*r* values < 0.10) and given the limitations of our sample for consideration of the number of covariates in the regression model, we did not include age. Regression model assumptions—linearity, normality of residuals, and homogeneity of residuals—were plotted and visually inspected; no significant assumption violations were identified. Multicollinearity was also assessed using both Pearson's correlations (all r values of indicator variables in the same model ≤0.50; see Table 2) and the Variance Inflation Factor (all values < 1.3; five is indicative of severe multicollinearity) with no incidences of multicollinearity identified.

**TABLE 2:** Correlations for study variables.

Variable	1. Age	2. Threat	3. Deprivation	4. Cumulative exposure	5. Irritability, child report
1. Age	—				
2. Threat	*r* = −0.00, CI:[‐0.28,0.28]	—			
3. Deprivation	*r* = −0.06, CI:[‐0.34,0.23]	**r = 0.50** ^b^, CI:[0.25,.69]	—		
4. Cumulative exposure	*r* = −0.03, CI:[‐0.31,0.26]	**r = 0.86** ^b^, CI:[0.77,.92]	**r = 0.87** ^b^, CI:[0.77,.92]	—	
5. Irritability, child report	*r* = −0.15, CI:[‐0.41,0.14]	*r* = 0.40^a^, CI:[0.13,0.61]	*r* = 0.26, CI:[‐0.03,0.51]	*r* = 0.38^a^, CI:[0.11,0.60]	—
6. Irritability, parent report	*r* = 0.08, CI:[‐0.20,0.40]	*r* = 0.35^a^, CI:[0.07,0.57]	*r* = 0.07, CI:[‐0.22,0.34]	*r* = 0.24, CI:[‐0.05,0.49]	**r = 0.52** ^b^, CI:[0.28,.70]

*Note*: statistics reflect uncorrected *p* values and 95% confidence intervals (CI). **Bolded** associations reflect those that survived correction for multiple comparisons.

^a^ *p* < .05.

^b^ *p* < .01.

### Data analysis

Primarily, we tested relations between trauma (threat, deprivation, and cumulative exposure) and child‐reported irritability. We inspected the correlation matrix to assess the general structure of the data. Similarly, Welch's two sample *t*‐tests and one‐way ANOVAs were used to determine whether any variables of interest differed based on gender, race, or ethnicity. To test our hypotheses, we first fit simple linear regression models with each composite exposure variable (cumulative, threat, or deprivation) individually added as the independent variable and child‐reported ARI total score as the dependent variable in separate models. Then, we fit a model including both threat and deprivation to investigate unique effects of exposure type on irritability. These methods were replicated using parent‐report data and are presented in the Supplementary Results. Sensitivity analyses are presented in the Supplementary Materials.

## RESULTS

Demographic characteristics and descriptive statistics are detailed in Table 1. Age was not associated with child‐reported severity of symptoms nor any measure of exposure to adversity (Table 2). Welch's two sample *t*‐test indicated that girls reported significantly greater irritability than boys, *t*(42.85) = 2.31, *p* = 0.026, *d* = 0.71, 95% CIs_std_ = [LLCI1: −1.32, ULCI2: −0.09]. Girls and boys did not report significantly different threat, deprivation, or cumulative exposure (see Supplementary Tables 2 and 3). Most of the sample (*n* = 32/48) identified as Hispanic/Latin. There were no differences in independent nor dependent variables based on race nor ethnicity (see Table 3 and Supplementary Tables 4 and 5). Consistent with previous publications (Hodgdon et al., 2021), results based on youths' self‐reported irritability are described herein (Dougherty et al., 2021). Corresponding analyses regarding parent‐reported irritability are reported in the Supplementary Materials (Supplementary Figures 4‐6; Supplementary Table 6).

**TABLE 3:** Welch's two sample *t*‐tests to compare gender differences.

	*t*	*p*	Cohen's *d*	LLCI^a^ (2.5%)	ULCI^a^ (97.5%)
Threat	−1.23	0.376	−0.37	−0.97	0.23
Deprivation	−0.22	0.828	−0.06	−0.65	0.52
Cumulative exposure	−0.82	0.416	−0.25	−0.83	0.34
**Irritability, child report**	**−2.31**	**0.026**	**−0.71**	**−1.32**	**−0.09**
Irritability, parent report	−1.31	0.196	−0.39	−0.97	0.20

*Note*: Bolded values highlight significant effects.

^a^ LLCI = lower level confidence interval; ULCI = upper level confidence interval. 95% CIs for Cohen's *d* are provided.

### Hypothesis 1: Cumulative exposure is associated with irritability

**FIGURE 1:**
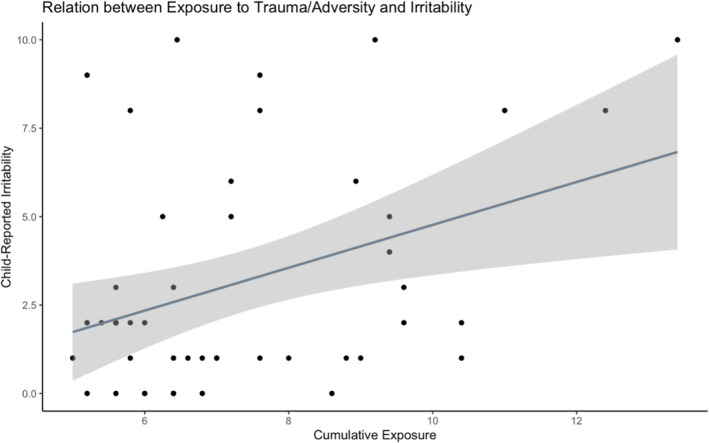
Cumulative exposure to trauma/adversity was associated with greater self‐reported irritability, and this effect remained significant after removing values of excessive influence (see Supplementary Figure 1).

There was a significant, positive effect of cumulative exposure on irritability, such that youth who had higher average CTQ scores reported more severe irritability, *F*(1,46) = 7.76, *B*_std_ = 0.38, *R* ^*2*^_adj_ = 0.13, *p* = 0.008, 95% Confidence Intervals (CIs)_std_ = [LLCI: 0.13; ULCI: 0.63] (Figure 1). Given that irritability significantly differed between boys and girls, we then adjusted for gender and found that the effect of cumulative exposure on irritability (*B*_std_ = 0.35, *t* = 2.62, *p* = 0.012, 95% CIs_std_ = [LLCI: 0.10, ULCI: 0.59]) remained significant after adjusting for gender (*B*_std_ = 0.27, *t* = 2.047, *p* = 0.047, 95% CIs_std_ = [LLCI: 0.02, ULCI: 0.61]); overall model *F*(2,45) = 6.24, *R* ^*2*^_adj_ = 0.18, *p* = 0.004. Findings corrected for values of excessive influence based on Cook's distance also indicated a significant effect of cumulative exposure on irritability (*B*_std_ = 0.39, *t* = 2.81, *p* = 0.007, 95% CIs_std_ = [LLCI: 0.14, ULCI: 0.64]) but not gender (*B*_std_ = 0.19, *t* = 1.35, *p* = 0.185, 95% CIs_std_ = [LLCI: −0.08, ULCI: 0.45]); overall model *F*(2,42) = 5.31, *R* ^*2*^_adj_ = 0.16, *p* = 0.009 (see Supplementary Figure 1). To summarize, our first hypothesis that cumulative exposure is associated with irritability was supported. Cumulative exposure explained significant variance in severity of self‐reported irritability in youth, above and beyond the effect of gender.

### Hypothesis 2: Unique effects of threat versus deprivation on irritability

**FIGURE 2:**
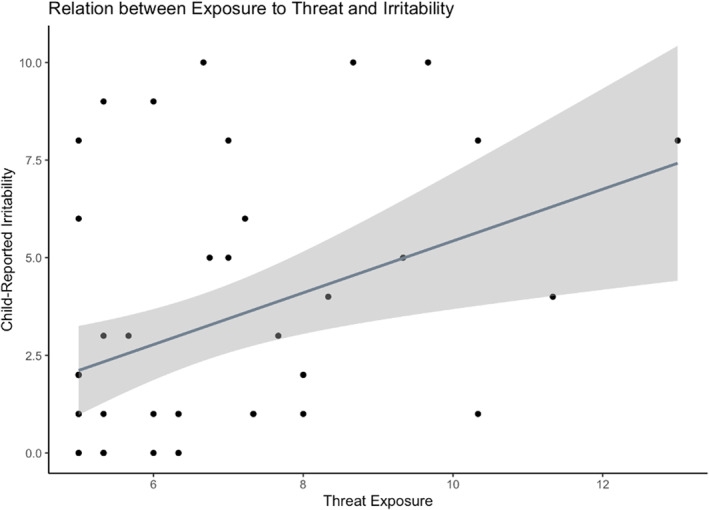
There was a significant effect of exposure to threat on child‐reported severity of irritability, and this effect remained significant after removing values of excessive influence (see Supplementary Figure 2).

There was a significant, positive association between threat and irritability, such that greater threat exposure was correlated with more severe self‐reported irritability, *F*(1,46) = 8.64, *B*_std_ = 0.40, *R* ^*2*^_adj_ = 0.14, *p* = 0.005, 95% CIs_std_ = [LLCI: 0.15, ULCI: 0.64] (see Figure 2 and Supplementary Figure 2). The relation remained significant when adjusting for gender (threat: *B*_std_ = 0.35, *t*(45) = 2.91, *p* = 0.011, 95% CIs = [LLCI: 0.11, ULCI: 0.60]; gender: *B*_std_ = 0.25, *t*(45) = 2.00, *p* = 0.067, 95% CIs = [LLCI: −0.00, ULCI: 0.50]; overall model *F*(2,45) = 6.33, *R* ^2^_adj_ = 0.18). Findings corrected for values of excessive influence based on Cook's distance also indicated a significant effect of threat exposure but not gender on irritability (see Supplementary Figure 2).

**FIGURE 3:**
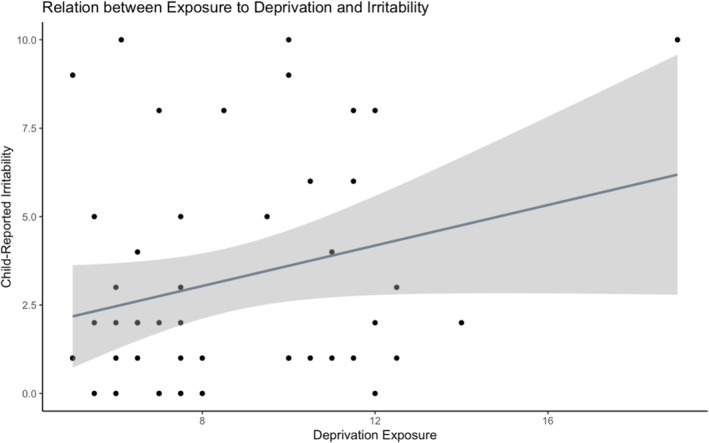
There was not a significant effect of exposure to deprivation on child‐reported severity of irritability, and this effect further diminished after removing values of excessive influence (see Supplementary Figure 3).

There was not a significant association between deprivation and irritability, *F*(1,46) = 3.35, *B*_std_ = 0.26, *R* ^*2*^_adj_ = 0.05, *p* = 0.074, 95% CIs_std_ = [LLCI: −0.01, ULCI: 0.53] (see Figure 3 and Supplementary Figure 3). This effect remained insignificant when adjusting for values of excessive influence (see Supplementary Figure 3). Given that the effect of deprivation on irritability was not significant, we did not further test gender as a covariate.

Finally, threat and deprivation, along with gender, were included in the same model to query unique effects of each type of exposure when adjusted for the other. Given that threat and deprivation were correlated with one another (*r*(46) = 0.50), independent variables were grand mean centered. We found that threat, adjusted for deprivation, was significantly associated with severity of irritability when controlling for gender (see Table 4). The effect of deprivation on irritability was not significant. The specific relation between threat and irritability, but not deprivation and irritability, was replicated in analyses based on parent‐reported irritability, see Supplementary Table 3. In sum, our second hypothesis regarding unique effects of threat and deprivation on irritability was supported. Threat exposure explained significant variance in severity of irritability in youth, above and beyond the effect of gender. However, deprivation did not explain significant variance in irritability.

**TABLE 4:** Multivariate regression: Effects of threat, deprivation, and gender on irritability.

Effect	Standardized estimate	Standardized *SE*	Standardized 95% CI		*t*	*p*
			*LL*	*UL*		
Threat	0.35	0.14	0.06	0.64	2.45	0.019
Deprivation	0.04	0.15	−0.26	0.33	0.24	0.811
Gender^a^	0.20	0.13	−0.06	0.47	1.55	0.129

*Note*: The overall model was significant, F(3,42) = 3.50, R^2^_adjusted_ = 0.14, *p* = 0.024. CI = confidence interval; *LL* = lower limit (2.5%); *UL* = upper limit (97.5%); SE = standard error (here, standardized). Data are corrected for values of excessive influence detected using Cook's distance. Estimates reflect standardized coefficients.

^a^ 0 = Male; 1 = Female.

## DISCUSSION

Traumatic early life experiences are estimated to explain ∼30% of child and adult psychopathology (Kessler et al., 2010); therefore, it is fitting to consider the role of threat, deprivation, and cumulative adverse early life experiences in the development, presentation, maintenance, severity, of irritability. The present study provides preliminary evidence that cumulative exposure is associated with irritability. This finding is in line with a broader literature highlighting that cumulative exposure to adverse and traumatic events confers increased risk for negative health behaviors (e.g., smoking), physical inactivity, psychopathology (e.g., depression, anxiety, posttraumatic stress), suicidality and attempts, and negative physical health conditions (e.g., cardiometabolic diseases) (Felitti et al., 1998; Gillespie et al., 2009). However, given that exposure to negative early life events is associated with a plethora of negative health outcomes and functional impairments, it may be possible that the associations described herein between be driven or enhanced by other co‐occurring psychological or physical symptoms.

While replicating designs from critical previous research is important to conceptually link negative early life experiences and irritability in this preliminary study, a growing body of work has also highlighted the limitations of grouping exposure types together and called for greater nuance in exploring the potential unique effects of different exposure types (Busso et al., 2017; Hinchey et al., 2023a, 2023b; Machlin et al., 2019; McLaughlin et al., 2014; McLaughlin & Sheridan, 2016; Miller et al., 2018; Sheridan & McLaughlin, 2014). One example of variant exposure types is threat versus deprivation. In our analyses, we found that threat, but not deprivation, drove the relation between negative exposures and irritability.

### Differential associations with irritability and threat versus deprivation

Irritability has previously been conceptualized as a maladaptive approach response to threat (Brotman, Kircanski, Stringaris, et al., 2017; Panksepp, 2006), and therefore it is not surprising that we see a specific association between threat and irritability in the present sample. Previous work has indicated that emotional learning may be differentially affected by exposure types, whereby children exposed to threat are more likely to show differences in fear learning compared to non‐exposed youth, and children exposed to deprivation are more likely to show differences in reward learning, compared to non‐exposed youth (Grasser & Jovanovic, 2021; McLaughlin & Sheridan, 2016). Such differences in fear and reward learning may also confer differential risk for psychopathology. For example, disrupted fear learning has been associated with anxiety disorders, and irritability is a common feature of pediatric anxiety (Brotman, Kircanski, Stringaris, et al., 2017; Stoddard et al., 2014, 2017). Irritability may reflect aberrant approach responses to threatening contexts given the presence of negative stimuli or scenarios. Mounting evidence, including our present findings, indicates that experiences classified as ‘threat’ (e.g., abuse) are likely to impact emotional learning, increase vigilance and arousal, and increase risk for both internalizing and externalizing psychopathology. Conversely, deprivation (e.g., neglect, poverty) reflects the absence of expected positive input that facilitate healthy development, potentially eliciting distinct responses from threat in response to the lack of reward. Here, growing evidence indicates negative effects of deprivation on cognition, cortical development, increased risk for internalizing psychopathology and language deficits.

### Sex differences

Evidence to date shows mixed findings for the effects of sex and gender on irritability (Humphreys et al., 2019; Singh & Wendt, 2024; Vidal‐Ribas et al., 2023). This may be driven by the variant indices used to quantify irritability and reporter bias, among other factors. For example, while parents may report higher levels of irritability in male youth, female youth self‐report more severe irritability (Vidal‐Ribas et al., 2023). In our sample, severity of irritability based on child self‐report was also greater in females compared to males. The difference in parent‐reported irritability was not statistically significant for males versus females, however the mean of parent‐reported irritability was slightly higher for male youth compared to female youth. Despite these sex differences, the relation between cumulative trauma and irritability, as well as threat exposure and irritability, remained significant when controlling for the effect of sex. Some studies have found a lack of sex or gender differences in the prevalence of irritability. Even in the absence of such differences in severity of irritability, sex may moderate associations between irritability or aggression and internalizing/externalizing symptoms (Humphreys et al., 2019; Lee et al., 2023), as well as relations between trauma‐related outcomes and psychopathology more broadly (Klein & Corwin, 2002; Kofman et al., 2024). In the present study, we were unable to model a moderation effect of gender due to the small sample. This limitation signals a need for future research to directly test the interaction effects of sex and gender on the relation between threat exposure and irritability.

### Summary

Irritability in the aftermath of threat exposure may be an acute symptom, correspond with/contribute to development of future psychopathology, or may be resultant of other trauma‐related symptoms. Regardless, these data point toward the intersection between two important psychiatric phenotypes that have been underexplored in the literature and may have important implications for treatment, namely transdiagnostic approaches.

### Limitations

The main limitation of the present study was the modest sample size. However, this sample importantly stands in contrast to most of the irritability literature that relies heavily on non‐representative white samples. This sample helps contribute to making the evidence more generalizable and provides pilot data for further research exploring more nuanced questions regarding links between childhood exposures, irritability, other psychopathology, and treatment outcomes. However, our study is not sufficiently powered to make inferences about specific demographic groups. Additionally, these data provide preliminary evidence to support novel lines of research linking negative early life experiences with trauma and adversity to transdiagnostic clinical presentations such as irritability. Importantly, while participants reported past exposures and current irritability, this study is cross‐sectional and thus causal conclusions cannot be made, especially given that current irritability may bias reports of prior trauma exposure.

Retrospective reporting may also result in variant reporting due to recall bias. For example, Kapp et al. highlight how recent stressors may increase recollections of negative early life events, contributing to distress and poorer mental health (Kapp et al., 2022). Their study highlights the tenuous nature of retrospective data collection that could be influenced by the current emotional/contextual state of the reporter. Our study benefited from multiple reporters (caregiver and child) which may circumvent the issue of state‐based variation in recall, however this was not directly measured in the present study. Future longitudinal studies may build on this line of research by querying differences in the presentation of irritability (e.g., temper outbursts vs. irritable mood) by type and timing of exposures. Exposure and irritability were self‐report, and individuals may have over‐ or under‐reported due to motivations unmeasured and unknown by the research team, but possibly with the aim of increasing likelihood of receiving services or due to concerns regarding confidentiality and possible impact on caregivers or other individuals the child interacts with. Associations between self‐reported scales, here CTQ and ARI, may be inflated by within informant effect. Two results reduce this concern as a confound. There are differential associations between CTQ subscales and ARI, and the same pattern is found for cross informant report (parent ARI with self‐reported CTQ). Of note, the ARI (Affective Reactivity Index; measure used in our study to assess irritability) does not provide a clinically meaningful threshold, and clinical diagnoses were not made for individuals in the current sample. Therefore, interpretations regarding clinically meaningful thresholds or benchmarks cannot be extrapolated. Finally, these data derive from a sample of treatment‐seeking youth, and therefore the design may bias outcomes in capturing youth at either ends of the spectrum—those who may experience greater exposure and/or symptom severity, driving them/their caregivers to seek out care, or those who experience less exposure and/or functional impairment, thereby perhaps making accessing care more likely.

### Clinical and public health implications

Our data continue to support the notion that experience matters, and that individuals' experiences should be considered in clinical care. Numerous public health efforts have encouraged screening for traumatic experiences in clinical and educational settings, and our data underscore the importance of this to guide early intervention and prevent or reduce the impact of psychopathology. While a number of research studies focused on the characterization and treatment of irritability exclude for high levels of trauma exposure and comorbid posttraumatic stress disorder, this might result in a lack of understanding regarding critical etiological contributors to irritability (Grasser, Erjo, et al., 2023; Naim et al., 2021, 2023). Considering the role of trauma exposure in the etiology of irritability may help inform novel, personalized, and transdiagnostic treatments for youth with clinically impairing irritability. Additionally, given the comorbid nature of irritability with other psychopathology, including trauma‐related disorders like PTSD and anxiety disorders, it would be important for clinical research to re‐consider the inclusion of such individuals in clinical trials. Indeed, exposure‐based cognitive behavioral therapy approaches may be well‐suited to address both irritability and comorbid PTSD or anxiety in youth (Grasser, Erjo, et al., 2023; Naim et al., 2023). Finally, future research should test transdiagnostic treatments for trauma‐exposed youth who may not meet full diagnostic criteria for PTSD, but may be experiencing other clinically‐impairing symptoms as a result thereof and may benefit from trauma‐informed interventions such as exposure‐based treatments and cognitive restructuring.

## AUTHOR CONTRIBUTIONS

**Lana Ruvolo Grasser:** Conceptualization, Formal analysis, Visualization, Writing – original draft. **Ruiyu Yang:** Data curation, Formal analysis, Writing – review & editing. **Melissa A. Brotman:** Conceptualization, Supervision, Writing – review & editing. **Jillian Lee Wiggins:** Data curation, Funding acquisition, Investigation, Methodology, Resources, Writing – review & editing.

## CONFLICT OF INTEREST STATEMENT

The authors have declared no competing or potential conflicts of interest.

## Ethical considerations

Procedures were approved by the University of California, San Diego Institutional Review Board and were accepted by joint agreement at San Diego State University. Parents gave written informed consent and permission for their children to participate, and children provided written assent

## Supporting information

Supplementary Material

## Data Availability

Due to the sensitivity of the data, a redacted, deidentified dataset is available upon written, reasonable request to Dr. Wiggins for non‐commercial, research purposes only.
