# Supplementary material for: The contribution of childhood trauma to irritability symptoms
Source: JCPP Adv. 2024 Jul 1;5(1):e12260. doi: 10.1002/jcv2.12260 (PMC11889654; doi:10.1002/jcv2.12260)
Supplement: Supplementary file 1 — Supplementary Material [file JCV2-5-e12260-s001.docx]

**Supplementary Methods**

Cronbach’s alpha is a measure of the shared variance among items that make up a scale/instrument, with greater reliability (higher alpha value) reflecting more covariance between items relative to the variance. While the CTQ measures discreet events that are not necessarily intrinsically linked to one another, nonetheless, we here report Cronbach’s alpha for the total CTQ score (alpha=0.75), the threat subscale (alpha=0.84), and the deprivation subscale (alpha=0.79).

*Sensitivity Analyses*

To assess the robustness of findings by systematically varying methodological choices, we performed sensitivity analyses to address potential sources of bias or uncertainty. Primarily, we sought to examine the relation between negative early life experiences (threat and deprivation) and irritability by coding exposure to threat, deprivation, or any early life experience as three dichotomous variables (0=no exposure; 1=exposure of any frequency or severity). Secondarily, we also explored linear regression models with sex included as a covariate, for each of the five subtypes described by the CTQ as indicator variables in separate models.

**Supplementary Results: Main Analyses**

**Supplementary Table 1**

*Welch’s Two Sample t-tests to compare differences by cohort*

| Measure | *t* | *p* | Cohen’s d | LLCI^a^ (2.5%) | ULCI^a^ (97.5%) |
| --- | --- | --- | --- | --- | --- |
|  |  |  |  |  |  |
| Age | 1.40 | .173 | .56 | -0.24 | 1.35 |
| Threat | 0.09 | .928 | .03 | -0.65 | 0.71 |
| Deprivation | -1.00 | .321 | -.30 | -0.90 | 0.29 |
| Cumulative Exposure | -0.50 | .618 | -.16 | -0.77 | 0.46 |
| Irritability |  |  |  |  |  |
| Child Report | -1.81 | .077 | -.57 | -1.20 | 0.06 |
| Parent Report | -0.87 | .389 | -.27 | -0.89 | 0.35 |

^a^ LLCI = lower level confidence interval; ULCI = upper level confidence interval. 95% CIs for Cohen’s d are provided.

*Exploratory Item-Level Analyses*

While we did not find a difference in overall rates of exposure nor rates for threat or deprivation in girls compared to boys, previous literature has cited higher rates of sexual abuse in girls, with implications for sex differences in mental and physical health outcomes. Therefore, we used Welch’s Two Sample t-tests to investigate gender differences in exposure types and individual exposures. We identified that a marginal difference in frequency and severity of emotional abuse was reported in girls (*M*=9.23) compared to boys (*M*=7.27), t(40.95)=-1.97, p=.055, d=-.62, LLCI:-1.24, ULCI:0.01. There were no other differences in frequency and severity of physical abuse, sexual abuse, emotional neglect, nor physical neglect in girls compared to boys (**Supplementary Table 2**).

**Supplementary Table 2**

*Welch’s Two Sample t-tests to compare gender differences*

|  | Boys | | Girls | | Full Sample | | *t* | *p* | Cohen’s *d* |
| --- | --- | --- | --- | --- | --- | --- | --- | --- | --- |
|  | *M* | *SD* | *M* | *SD* | *M* | *SD* |  |  |  |
| Emotional Abuse | 7.27 | 2.47 | 9.23 | 4.29 | 8.33 | 3.67 | -1.97 | .055 | -0.62 |
| Physical Abuse | 6.22 | 2.88 | 5.66 | 1.36 | 5.92 | 2.18 | 0.84 | .406 | 0.31 |
| Sexual Abuse | 5.39 | 1.46 | 5.96 | 2.52 | 5.70 | 2.10 | -0.97 | .338 | -0.30 |
| Emotional Neglect | 9.64 | 3.54 | 9.85 | 5.06 | 9.75 | 4.38 | -0.17 | .867 | -0.05 |
| Physical Neglect | 7.36 | 2.34 | 7.51 | 2.30 | 7.44 | 2.29 | -0.22 | .829 | -0.07 |

To further explore whether variation by gender in specific exposures was masked by cumulative scores, we used Spearman rank-order tests (see **Supplementary Table 3**) and found gender differences in frequency and severity of reporting on items 14 (“People in my family said hurtful or insulting things to me”), 22 (“I had the best family in the world”), and 25 (“I believe I was emotionally abused”). Such variation likely drove the gender difference in the emotional abuse category (see **Supplementary Table 2**).

**Supplementary Table 3**

*Correlations for Study Variables*

| CTQ Item | Gender |
| --- | --- |
| 1. | r=.07  CI:[-.22,.35] |
| 2. | r=-.07  CI:[-.35,.22 |
| 3. | r=.19  CI:[-.10,.45] |
| 4. | r=.07  CI:[-.22,.35] |
| 5. | r=-.03  CI:[-.31,.26] |
| 6. | r=.07  CI:[-.22,.35] |
| 7. | r=-.06  CI:[-.34,.23] |
| 8. | r=.17  CI:[-.12,.43] |
| 9. | r=.02  CI:[-.30,.27] |
| 10. | r=.18  CI:[-.45,.10] |
| 11. | r=-.04  CI:[-.33,.25] |
| 12. | r=-.13  CI:[-.41,.16] |
| 13. | r=-.88  CI:[-.36,.20] |
| 14. | r=.33*  CI:[.05,.56] |
| 15. | r=-.02  CI:[-.30,.26] |
| 16. | r=.10  CI:[-.19,.38] |
| 17. | r=-.02  CI:[-.30,.26] |
| 18. | r=-.05  CI:[-.33,.23] |
| 19. | r=.06  CI:[-.23,.34] |
| 20. | r=.28  CI:[-.01,.52] |
| 21. | r=.19  CI:[-.10,.45] |
| 22. | r=-.36**  CI:[-.59,-.09] |
| 23. | r=.12  CI:[-.18,.39] |
| 24. | r=.23  CI:[-.06,.49] |
| 25. | r=.30*  CI:[.01,.54] |
| 26. | r=-.03  CI:[-.32,.25] |
| 27. | r=.17  CI:[-.12,.43] |
| 28. | r=.11  CI:[-.18,.38] |

**p*<.05, ***p*=.01

**Supplementary Table 4**

*One-Way Analyses of Variance on Exposures and Symptom Severity by Race*

| Measure | *F*(4,34) | *p* | η^2^ |
| --- | --- | --- | --- |
|  |  |  |  |
| Threat | 1.00 | .419 | .11 |
| Deprivation | 0.12 | .973 | .01 |
| Cumulative Exposure | 0.54 | .710 | .06 |
| Irritability |  |  |  |
| Child Report | 0.53 | .712 | .06 |
| Parent Report | 0.72 | .582 | .08 |

**Supplementary Table 5**

*Welch’s Two Sample t-tests to compare differences by ethnicity (Hispanic/Latin compared to non-Hispanic/Latin)*

| Measure | *t* | *p* | Cohen’s d | LLCI^a^ (2.5%) | ULCI^a^ (97.5%) |
| --- | --- | --- | --- | --- | --- |
|  |  |  |  |  |  |
| Threat | -0.95 | .347 | -.34 | -1.05 | 0.37 |
| Deprivation | 0.41 | .687 | .13 | -0.50 | 0.76 |
| Cumulative Exposure | -0.34 | .733 | -.11 | -0.77 | 0.54 |
| Irritability |  |  |  |  |  |
| Child Report | 1.42 | .163 | .42 | -0.17 | 1.01 |
| Parent Report | -0.53 | .603 | -.20 | -0.92 | 0.53 |

^a^ LLCI = lower level confidence interval; ULCI = upper level confidence interval. 95% CIs for Cohen’s d are provided.


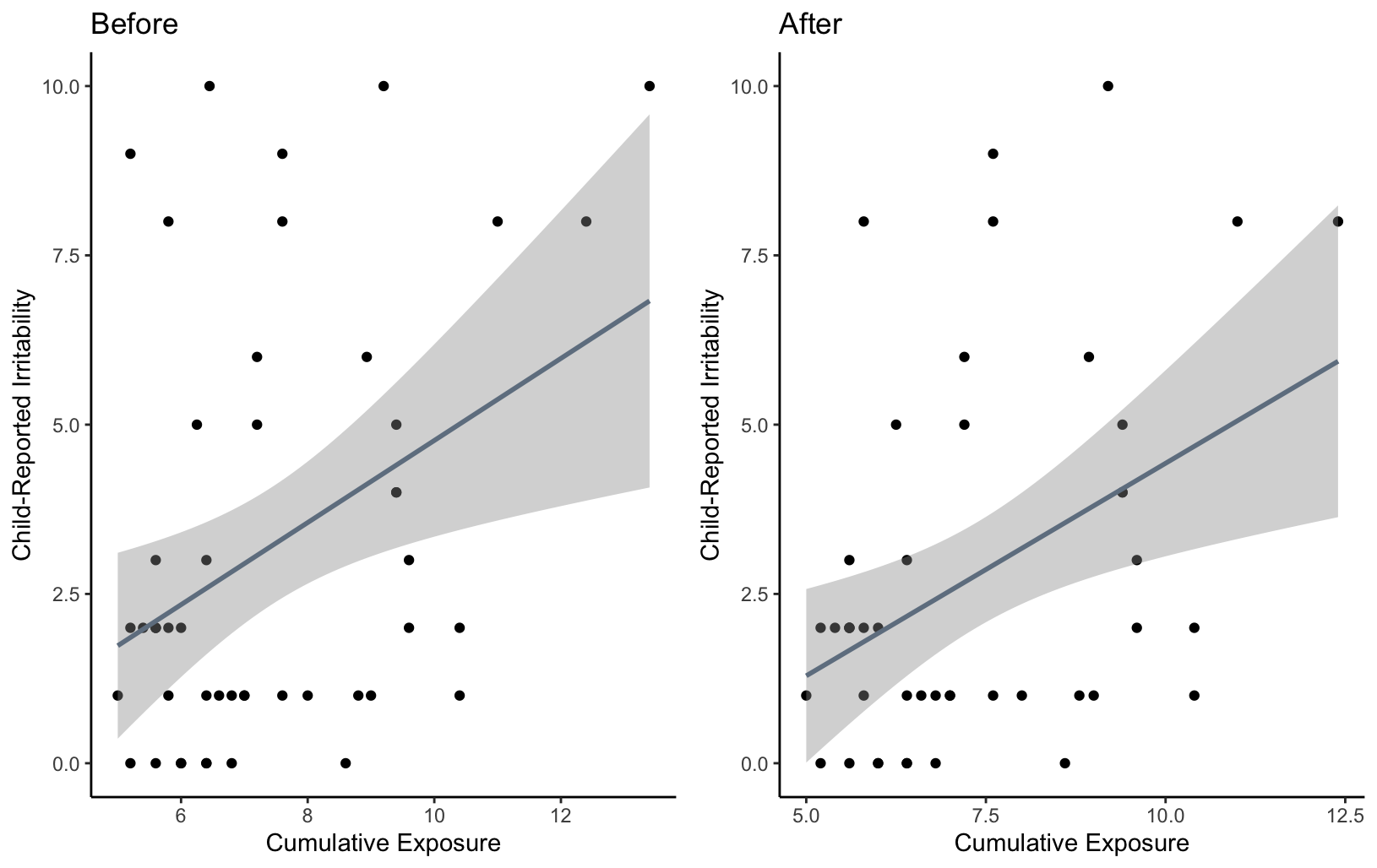


Supplementary Figure 1. Significant relation between cumulative exposure and child-reported irritability before (left) and after (right) deletion of excessively influential values.

**
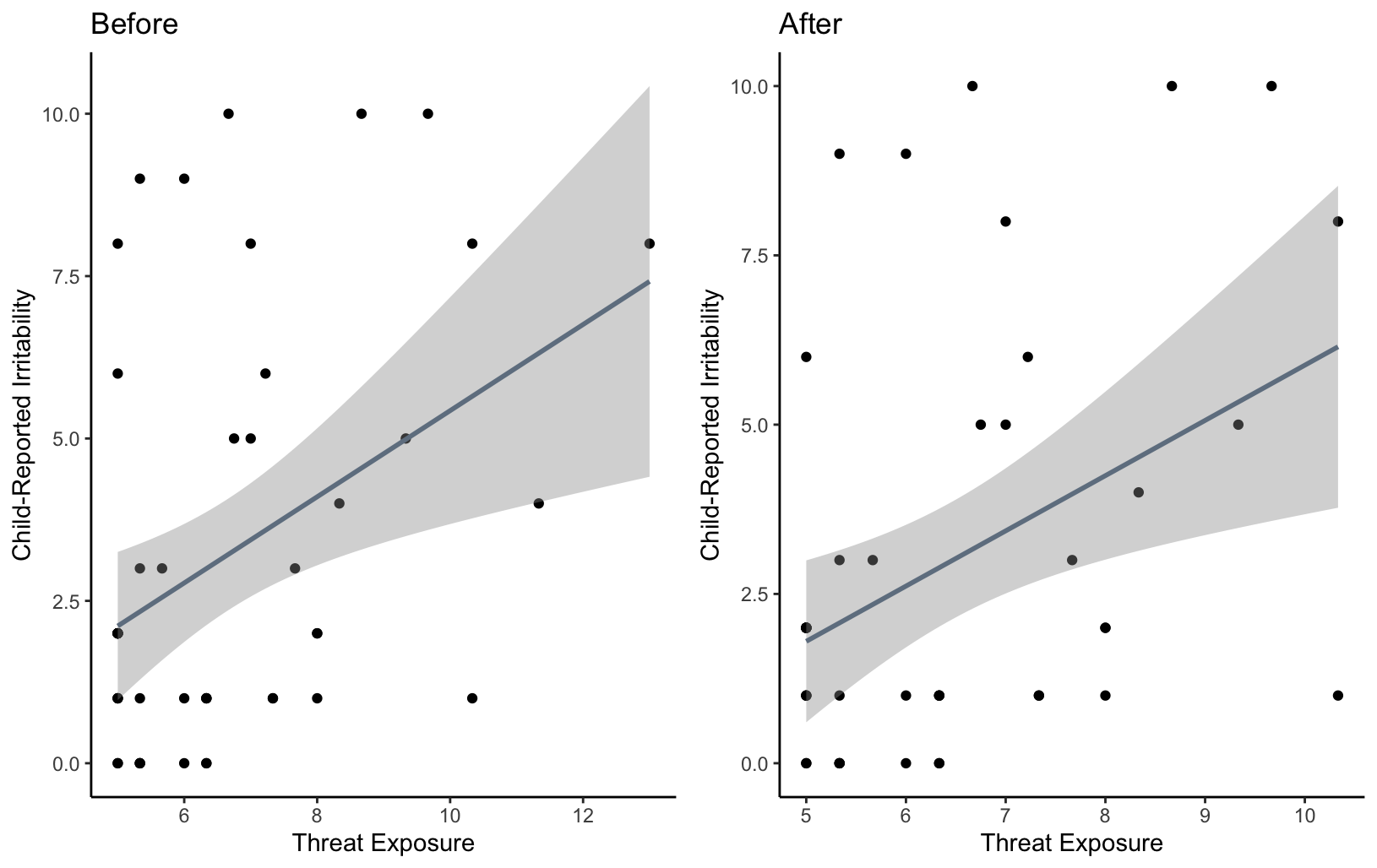
**

Supplementary Figure 2. Significant relation between threat exposure and child-reported irritability before (left) and after (right) deletion of excessively influential values. The model corrected for values of excessive influence indicated a significant effect of threat (B_std_=.40, p=.005, 95% CIs: [LLCI: 0.16, ULCI: 0.64]) but not gender (B_std_=.21, p=.126, 95% CIs: [LLCI: -0.05, ULCI: -.47]), overall model F(2,44)=7.02, p=.002, R^2^_adj_=.21.


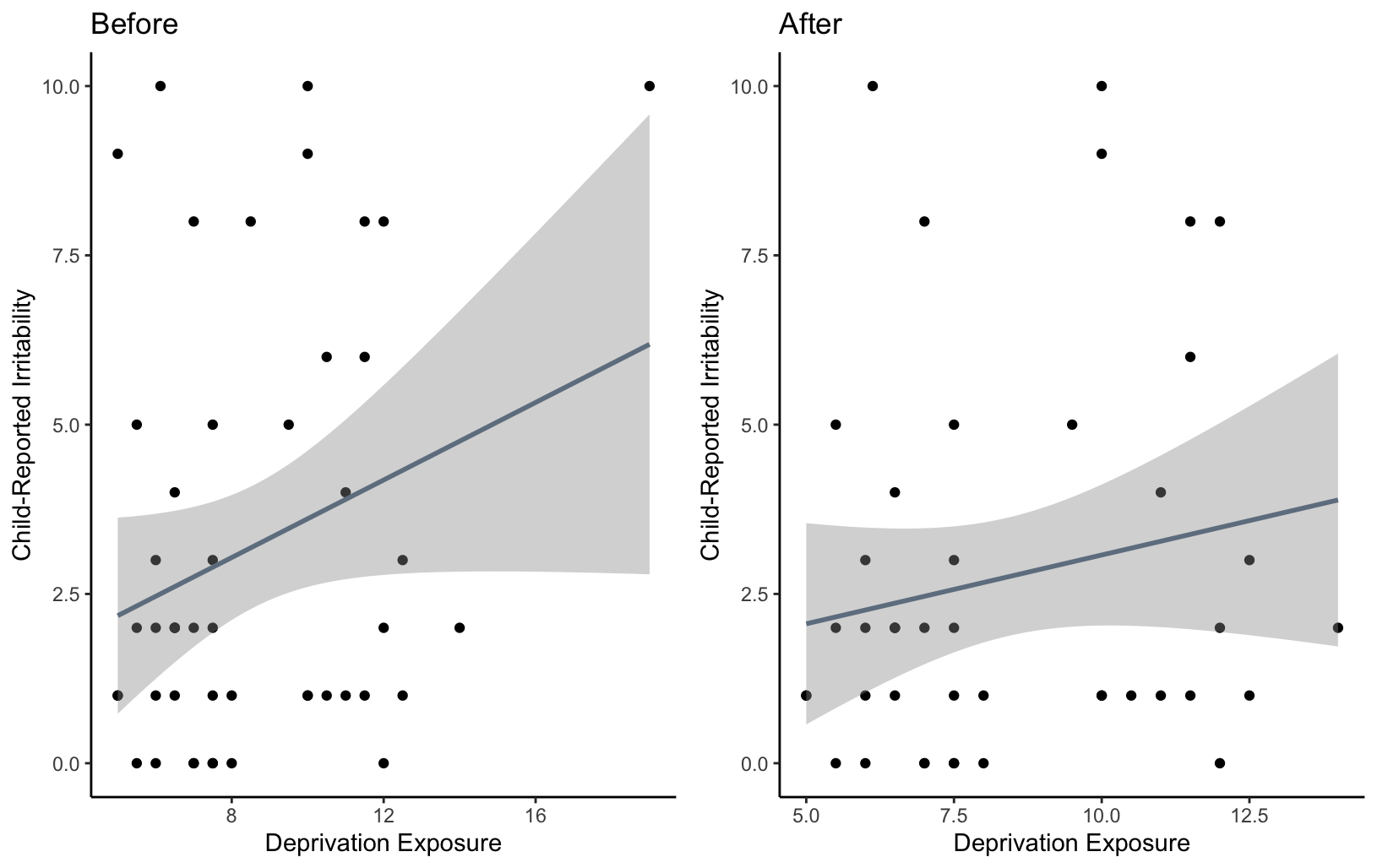


Supplementary Figure 3. Significant relation between deprivation exposure and child-reported irritability before (left) and after (right) deletion of excessively influential values. The model corrected for values of excessive influence confirmed the non-significant effect of deprivation, F(1,43)=3.08, B_std_=.26, p=.087, R^2^_adj_=.05, 95% CIs: [LLCI: -.02, ULCI: 0.54].

**Supplementary Results: Sensitivity Analyses**

Welch’s two sample t-test indicated that there was a moderate, non-significant effect of exposure to threat on irritability, *t*(37.79)=-1.68, *p*=.101, d=-0.55, 95% CIs: [LLCI: -1.19; ULCI: 0.11], such that individuals who reported any threat exposure (n=34) had on average greater irritability (*M*=3.62) than individuals who did not report any threat exposure (n=14; *M*=2.21).

Welch’s two sample t-test did not indicate an effect of exposure to deprivation on irritability, *t*(2.13)=0.18, *p*=.873, d=0.25, 95% CIs: [LLCI: -2.48, ULCI: 2.92]. Youth who reported any deprivation exposure (n=45) did not have greater irritability (*M*=3.18) than individuals who did not report any deprivation exposure (n=3; *M*=3.67).

Notably, most youth in this sample reported at least one exposure (threat or deprivation), and very few reported absence of deprivation exposure. Because of this, we were not able to dichotomously compare exposure to any negative early life experience with no exposure. Additionally, the results described above should be interpreted with caution, as this dichotomous approach was not part of our original hypotheses and is underpowered with extremely unbalanced groups. This supports the use of the continuous modeling presented in our main analyses and results.

Five multiple linear regression models with sex (male versus female) included as a covariate were performed to test each of the five CTQ subscales (emotional abuse, physical abuse, sexual abuse, emotional neglect, and physical neglect) as an indicator variable. The model including emotional abuse as the indicator variable was significant, *F*(2,45)=8.01, *R^2^_adj_*=0.23, *p*=.001. Emotional abuse explained a significant amount of variance in irritability when controlling for the non-significant effect of sex, *B*_std_=0.42, t(45)=3.57, *p*<.001, 95% CIs: [LLCI=0.18; ULCI=0.66]. There were no other significant effects of any CTQ subscales, all *p_s_>*.05. The regression analyses seem to indicate the specificity of threat exposure in explaining a significant portion of variance in irritability (confirming our main analyses) that may be driven by exposure to and frequency of emotional abuse.

**Supplementary Results: Parent-Report**

**Results**

*Hypothesis 1: Cumulative exposure is associated with irritability*

Child-reported cumulative exposure was not significantly associated with parent-reported severity of youth irritability, *F*(1,46)=2.74, *B*_std_=.24, *R*^2^_adj_=.04, *p*=.104, 95% CIs_std_=[LLCI: -0.04, ULCI: 0.51] (**Supplementary** **Figure 4**).


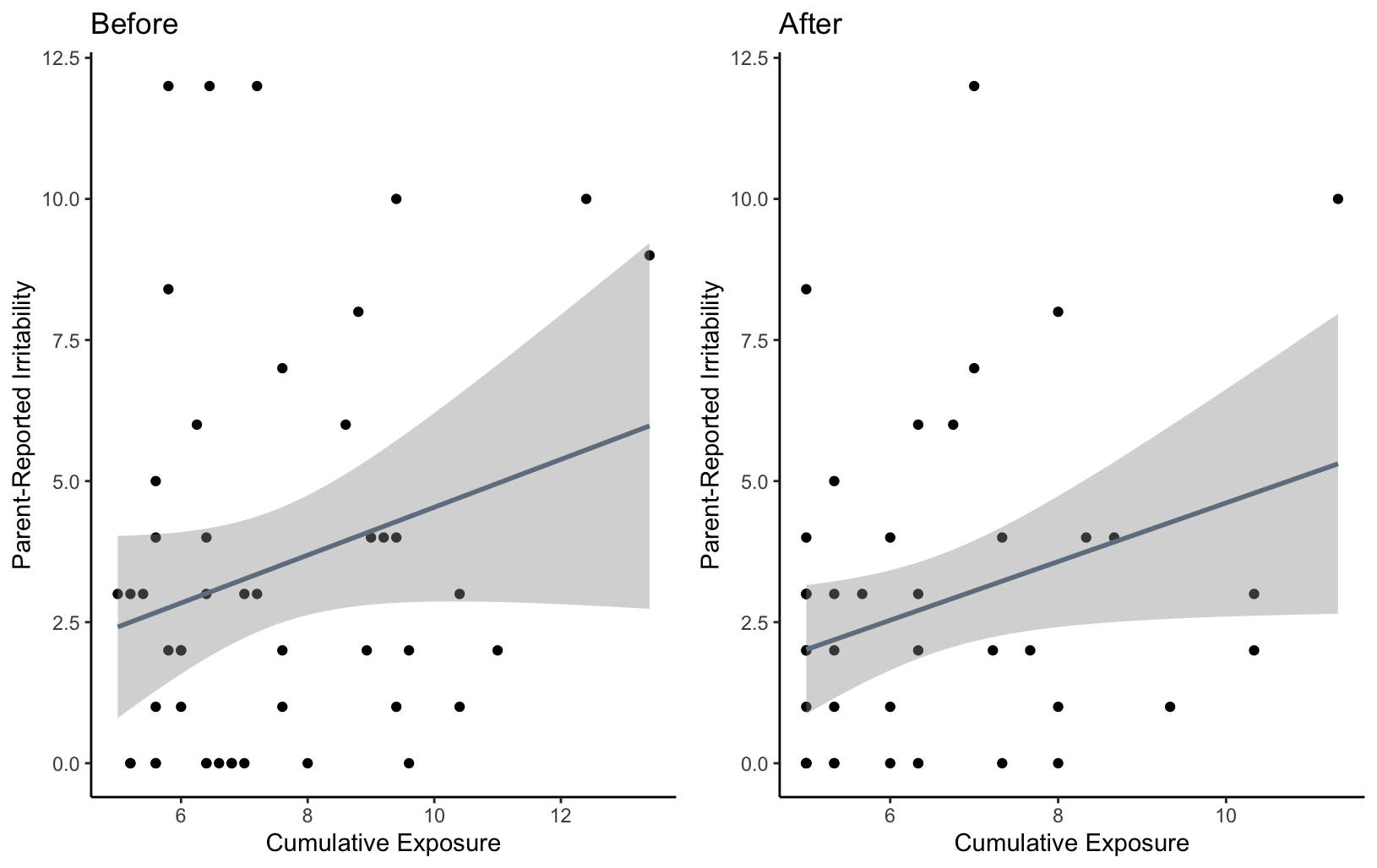


Supplementary Figure 4. Cumulative exposure to trauma/adversity was not associated with parent-reported irritability (left; before multivariate outlier correction), and the magnitude of this effect was further diminished after controlling for multivariate outliers (right), F(1,42)=0.92, B_std_=.15, R^2^_adj_=-.002, p=.344, 95% CIs_std_=[LLCI: -0.15, ULCI: 0.44].

*Hypothesis 2: Unique effects of threat versus deprivation on irritability*

There was a significant, positive association between threat and child’s irritability, such that greater threat exposure was associated with more severe parent-reported irritability, *F*(1,46)=6.25, *B*_std_=.35, *R*^2^_adj_=.10, *p*=.016, 95% CIs_std_=[LLCI: 0.09, ULCI: 0.60] (**Supplementary Figure 5**).


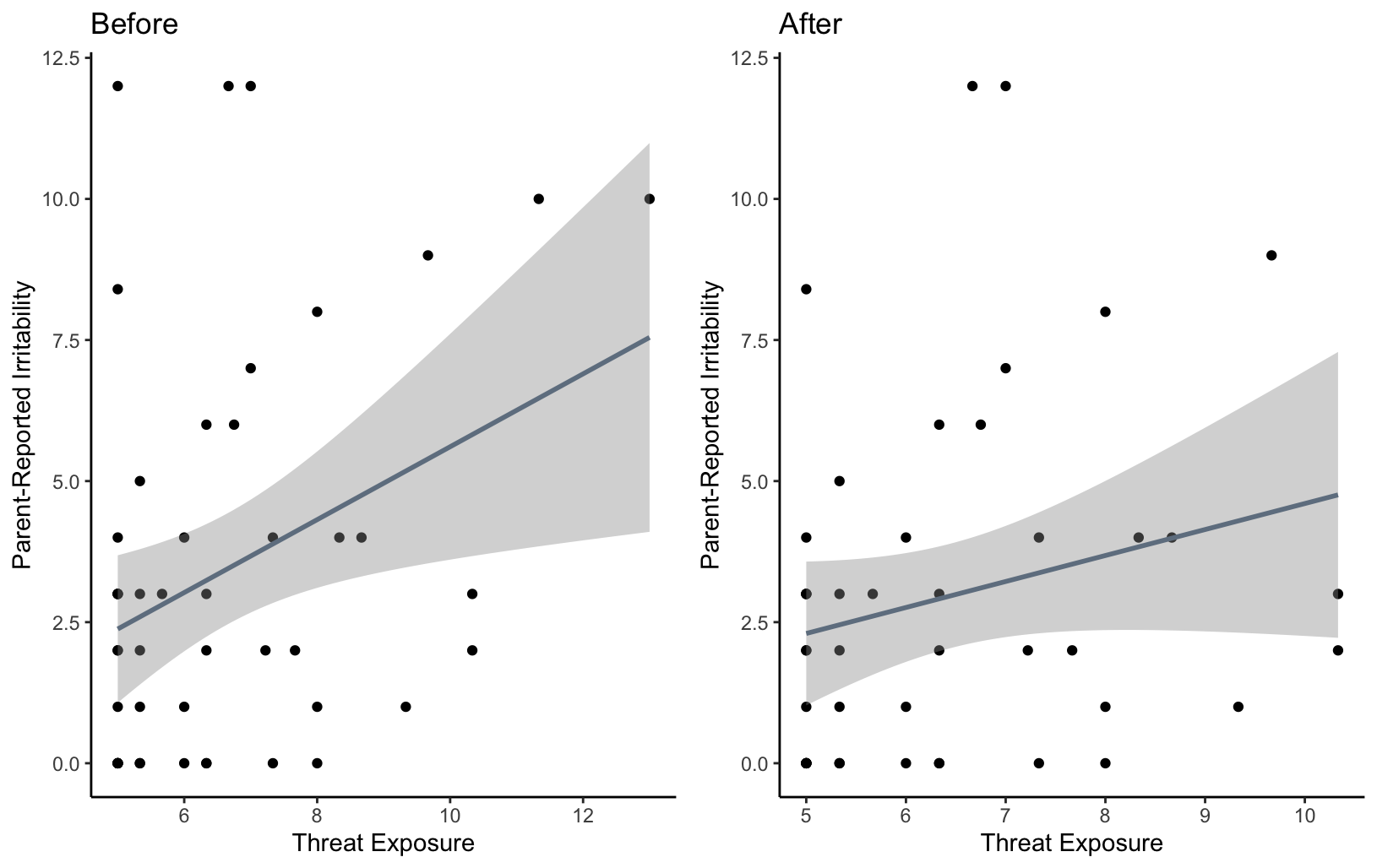


Supplementary Figure 5. The relation between child-reported exposure to threat and parent-reported severity of child’s irritability symptoms was significant, however this effect did not remain significant after removing multivariate outliers, F(1,43)=2.35, B_std_=.23, R^2^_adj_=.03, p=.133, 95% CIs_std_=[LLCI: -0.06, ULCI: 0.51].

There was not a significant association between deprivation and irritability, *F*(1,46)=0.21, *B*_std_=.07, *R*^2^_adj_=-.02, *p*=.653, 95% CIs_std_=[LLCI: -0.22, ULCI: 0.36] (**Supplementary Figure 6**).


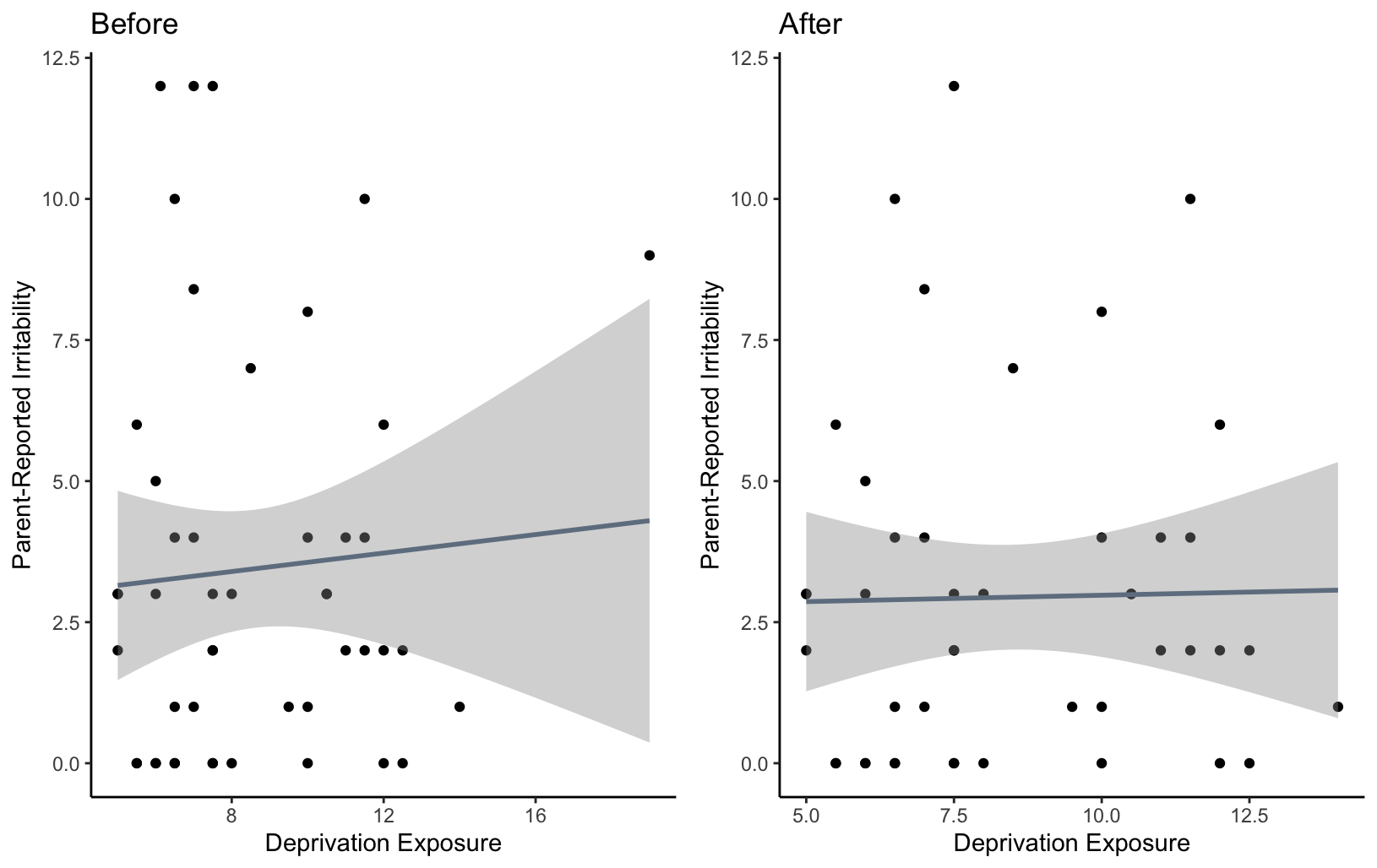


Supplementary Figure 6. Non-significant effect of child-reported exposure to deprivation on parent-reported severity of child’s irritability. The magnitude of the effect was further diminished when multivariate outliers were removed, F(1,43)=0.01, B_std_=.02, R^2^_adj_=-.02, p=.904, 95% CIs_std_=[LLCI: -0.28, ULCI: 0.32].

When threat and deprivation were entered into the same model, the relation between threat and parent-reported severity of child irritability remained significant when controlling for the effects of deprivation and gender (see **Supplementary** **Table 6**).

**Supplementary Table 6**

*Multivariate Regression: Effects of Threat, Deprivation, and Gender on Irritability. The overall model was significant, F(3,41)=4.05, R^2^=.17, p=.013.*

| Effect | Standardized Estimate | Standardized *SE* | Standardized 95% CI | | *p* |
| --- | --- | --- | --- | --- | --- |
|  |  |  | *LL* | *UL* |  |
| Threat | 0.50 | 0.14 | 0.22 | 0.77 | .003 |
| Deprivation | -0.24 | 0.15 | -0.53 | 0.06 | .092 |
| Gender^a^ | 0.11 | 0.13 | -0.16 | 0.38 | .455 |

*Note.* CI = confidence interval; *LL* = lower limit; *UL* = upper limit. Data are corrected for multivariate outliers. Data were corrected for values of excessive influence detected using Cook’s distance.

^a^ 0=Male; 1=Female
